# Combinatorial Recruitment of CREB, C/EBPβ and c-Jun Determines Activation of Promoters upon Keratinocyte Differentiation

**DOI:** 10.1371/journal.pone.0078179

**Published:** 2013-11-07

**Authors:** Julian M. Rozenberg, Paramita Bhattacharya, Raghunath Chatterjee, Kimberly Glass, Charles Vinson

**Affiliations:** 1 Department of Pathology and Lab Medicine, University of North Carolina at Chapel Hill, Chapel Hill, North Carolina, United States of America; 2 Crystallography and Molecular Biology Division, Saha Institute of Nuclear Physics, West Bengal, India; 3 Human Genetics Unit, Biological Science Division, Indian Statistical Institute, Kolkata, India; 4 Harvard School of Public Health, Dana-Farber Cancer Institute, Boston, Massachusetts, United States of America; 5 Laboratory of Metabolism, National Cancer Institute, Bethesda, Maryland, United States of America; University of Kansas School of Medicine, United States of America

## Abstract

**Background:**

Transcription factors CREB, C/EBPβ and Jun regulate genes involved in keratinocyte proliferation and differentiation. We questioned if specific combinations of CREB, C/EBPβ and c-Jun bound to promoters correlate with RNA polymerase II binding, mRNA transcript levels and methylation of promoters in proliferating and differentiating keratinocytes.

**Results:**

Induction of mRNA and RNA polymerase II by differentiation is highest when promoters are bound by C/EBP β alone, C/EBPβ together with c-Jun, or by CREB, C/EBPβ and c-Jun, although in this case CREB binds with low affinity. In contrast, RNA polymerase II binding and mRNA levels change the least upon differentiation when promoters are bound by CREB either alone or in combination with C/EBPβ or c-Jun. Notably, promoters bound by CREB have relatively high levels of RNA polymerase II binding irrespective of differentiation. Inhibition of C/EBPβ or c-Jun preferentially represses mRNA when gene promoters are bound by corresponding transcription factors and not CREB. Methylated promoters have relatively low CREB binding and, accordingly, those which are bound by C/EBPβ are induced by differentiation irrespective of CREB. Composite “Half and Half” consensus motifs and co localizing consensus DNA binding motifs are overrepresented in promoters bound by the combination of corresponding transcription factors.

**Conclusion:**

Correlational and functional data describes combinatorial mechanisms regulating the activation of promoters. Colocalization of C/EBPβ and c-Jun on promoters without strong CREB binding determines high probability of activation upon keratinocyte differentiation.

## Introduction

During differentiation, keratinocytes exit the cell cycle and start producing differentiation-specific proteins used in the formation of the outer skin layer. Both the c-Jun/AP-1 family of transcription factors [1], [2], [3], [4] and C/EBPs [5], [6], [7] play a pivotal role in the regulation of keratinocyte differentiation. Keratinocytes proliferation and oncogenic transformation is also dependent upon C/EBP’s [8]–[10] and c-Jun/AP-1 [11]–[17]. C/EBPβ, in particular, is known to be a positive regulator of keratinocyte proliferation [18], [8], [9]. C/EBPβ is activated by Ras and C/EBPβ-nullizygous mice are completely refractory to skin tumor development [9]. One of the mechanisms of C/EBPβ mediated resistance to skin carcinogenesis is repression of p53 [10], [18]. In contrast, C/EBPα is targeted by p53 [19], and blocks Ras-induced and epidermal growth factor-induced E2F activity in keratinocytes as well as Ras-induced cell transformation and cell cycle progression [20]. Both C/EBPα and C/EBPβ regulate genes involved in keratinocyte differentiation, including involucrin [7], keratin 1 and keratin 10 [6], [21], and desmocolin [22]. Mice lacking both of these proteins in the epidermis show increased proliferation of basal keratinocytes and impaired commitment to differentiation [6]. c-Jun/AP-1 deficiency augments keratinocyte resistance to carcinogenesis by mechanisms associated with the repression of AP-1 targets that promote proliferation such as Cyclin D1 [15], [16], [23] and EGFR [24].

The mechanism that allows for the selective regulation of genes involved in diverse cell functions such as proliferation and differentiation using the same transcription factors is still unknown. Differential expression of AP-1 family members c-Jun/JunB, Fra2/cFos and C/EBPs family members C/EBPα and C/EBPβ contribute to the modulation of gene activities upon differentiation [11], [22], [13], [25]. Since the DNA binding domains of these transcription factors within their families are identical [26], exchanges of the transcription factor bound to DNA will occur at the regulatory elements of genes that are (or are not) activated by differentiation. One possible explanation for selective activation of promoters by transcription factors is the binding of heterodimers of AP-1 and/or C/EBPβ to composite elements [27]–[30]. In this case, the c-Jun-C/EBPβ heterodimer represses transcription [31], while promoters bound by C/EBPβ alone can be activated [32].

CREB is another transcription factor important for keratinocyte proliferation [2], [33], [34], [23]. In different cell types CREB is bound to promoters of nearly the same set of genes responsible for cell survival and cell cycle progression [35]. Inhibition of CREB leads to the repression of skin tumor initiation in mice and repression of cell cycle progression [33]. The role of CREB in keratinocyte differentiation is not well studied, although it has been shown that inhibition of CREB by A-CREB dominant negative represses both CREB and AP-1 reporter activities [34]. While some studies reported that CREB protein level is induced by keratinocytes differentiation [34], other show that it is repressed [2].

Gene activation and repression mediated by the binding of a transcription factor can also be driven by cooperative interactions with other sequence specific transcription factors [36]–[42]. For example, CREB, C/EBPs and/or AP-1 cooperatively regulate the promoter activity of prointerleukin-1 beta [29], loricrine [2], cFos [43], CyclinD1 [23], and StAR [44].

Our previous work has demonstrated that DNA methylation is important for activation of some tissue specific genes involved in keratinocyte differentiation [45]. Notably, although methylation inhibits CREB binding to its consensus binding site, C/EBPβ and c-Jun can bind the methylated CREB binding site. Also, methylation of the consensus C/EBP DNA binding site increases its affinity to C/EBP [45]. Thus, the DNA methylation status of promoters may play a role in differential recruitment of CREB, c-Jun and C/EBPβ.

We hypothesized that CREB, c-Jun and C/EBPβ are functioning differently when they bind to promoters in different combinations. Because CREB is important for cell survival, we hypothesized that C/EBPβ and c-Jun regulate genes involved in cell survival and proliferation if they co-localize with CREB and regulate genes involved in differentiation when they do not. We test this hypothesis by analyzing how genome-wide promoter binding of transcription factors CREB, C/EBPβ and c-Jun in different combinations correlate with either the activation or repression of promoters and their target genes upon keratinocyte differentiation.

## Results

### Upon Keratinocyte Differentiation RNAP is Preferentially Recruited to Promoters bound by a Combination of C/EBPβ and cJun

In order to determine factors correlating with activation of promoters as measured by RNA polymerase II (RNAP) binding, we determined localization of three transcription factors – CREB, C/EBPβ and c-Jun, as well as RNAP, in proliferating and differentiated keratinocytes using chromatin immunoprecipitation followed by microarray hybridization. Despite close to 100% immunoprecipitation efficiency, c-Jun had lower DNA binding levels than the other proteins (Figure S1).

**Figure 1:**
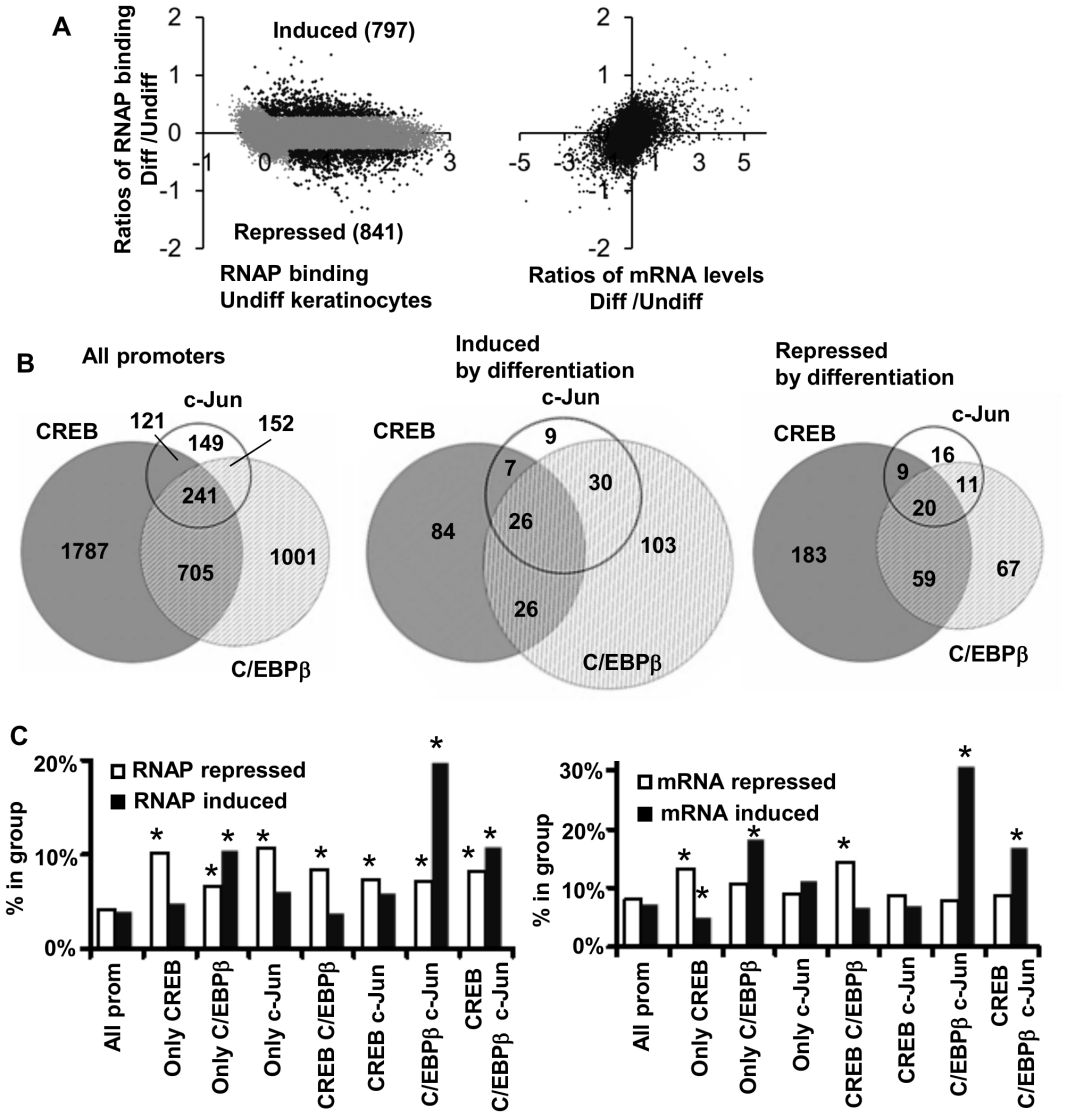
Upon keratinocyte differentiation RNAP binding and mRNA levels are preferentially induced when promoters are bound by combinations of C/EBPβ and cJun. **A.** Scatterplott of changes in RNAP binding after differentiation versus RNAP binding in undifferentiated keratinocytes show 797 and 841 promoters are induced or repressed upon differentiation. These changes correlate with changes in mRNA levels. **B.** Transcription factors CREB, C/EBPβ and c-Jun bind distinct set of promoters in differentiated keratinocytes. Euler diagrams show that promoters bound by C/EBPβ and c-Jun are preferentially induced by differentiation and promoters bond by CREB are not. **C.** Fraction of promoters bound by different combination of transcription factors in differentiated keratinocytes where RNAP binding is repressed (white bars) or induced by differentiation (black bars). **D.** Fraction of genes with mRNA levels induced or repressed by differentiation more than 1.4 times in groups of promoters bound by different combinations of transcription factors. * - values are different from expected (p<0.005 using a two-tailed unpaired t-test).

Upon keratinocyte differentiation, RNAP binding increased at 797 promoters and decreased at 841 promoters (Figure 1A). Out of 17928 genes for which we have both promoter binding data and mRNA expression levels, we observed that 1326 (7.4%) have increased mRNA levels and 1458 (8.1%) genes are repressed upon keratinocyte differentiation. This increase correlates with induction of RNAP binding (Figure 1A). In contrast, after keratinocyte differentiation the binding of CREB, C/EBPβ and c-Jun does not change dramatically (Figure S1A). The highest increase of binding was observed for, C/EBPβ (Figure S1 A, B). Scatterplots of changes in RNAP binding versus changes in transcription factor binding upon differentiation show that induction of RNAP upon differentiation is overrepresented among promoters with induction of C/EBPβ binding (Figure S1B). We did not observe this for c-Jun or CREB. Induction of RNAP is also preferentially observed in promoters bound by C/EBPβ and c-Jun, while promoters bound by CREB are predominantly repressed upon differentiation (Figure S1C). Thus, changes of RNAP binding upon differentiation are not determined only by binding of CREB, C/EBPβ or c-Jun to promoters.

We next analyzed if promoters bound by different combinations of transcription factors are preferentially repressed or induced upon differentiation. CREB, C/EBPβ and cJun bind distinct set of promoters in differentiated keratinocytes (Figure 1B). Among those, promoters bound by C/EBPβ and c-Jun are overrepresented in those which also have induction of RNAP binding upon differentiation (Figure 1B). Figure 1C shows the fraction of promoters bound by different combination of transcription factors in differentiated keratinocytes where RNAP binding or mRNA levels are repressed (white bars) or induced by differentiation (black bars). Induction of RNAP binding and mRNA by differentiation is the lowest for promoters bound by CREB alone or in combination with c-Jun or C/EBPβ, and the highest for C/EBPβ alone, C/EBPβ, cJun and CREB, C/EBPβ, c-Jun bound promoters. In particular, 20% of promoters bound by C/EBPβ and cJun are induced upon differentiation, five times more than expected by chance (Figure 1C).

We found the same absolute values of RNAP induction between groups (Figure S2A); however, induction of mRNA upon differentiation is highest when promoters are bound by c-Jun or C/EBPβ and lowest for genes with CREB-bound promoters (Figure S2 B).

Transcription factors are known to recruit histone acetyltransferases that change chromatin accessibility and transcriptional activation. Therefore, we next investigated whether H3K9 acetylation preferentially altered upon differentiation for promoters bound by specific combinations of transcription factors. As expected, H3K9 acetylation correlates with RNAP binding in differentiated (Figure S3A) and undifferentiated keratinocytes. Although, relatively wide range of RNAP binding for given H3K9 acetylation level suggests that RNAP binding is also regulated by other factors. Likewise, changes in RNAP binding upon differentiation positively correlate with changes in H3K9 acetylation (Figure S3B). We found 800 promoters with induced H3K9 acetylation and 186 that also have induced RNAP binding upon differentiation. Similar to what we observed for RNAP binding, H3K9 acetylation is preferentially induced on promoters bound by C/EBPβ and cJun and promoters bound by CREB, cJun and C/EBPβ (Figure S3C). Promoters bound by C/EBPβ and CREB and promoters bound by cJun and CREB have lower levels of H3K9 acetylation induction compared to promoters bound by C/EBPβ and c-Jun (Figure S3D).

### Colocalization of C/EBPβ or c-Jun with CREB Determine Genes which Expression and Induction upon Differentiation is Dependent on C/EBPβ and c-Jun Functions

**Figure 2:**
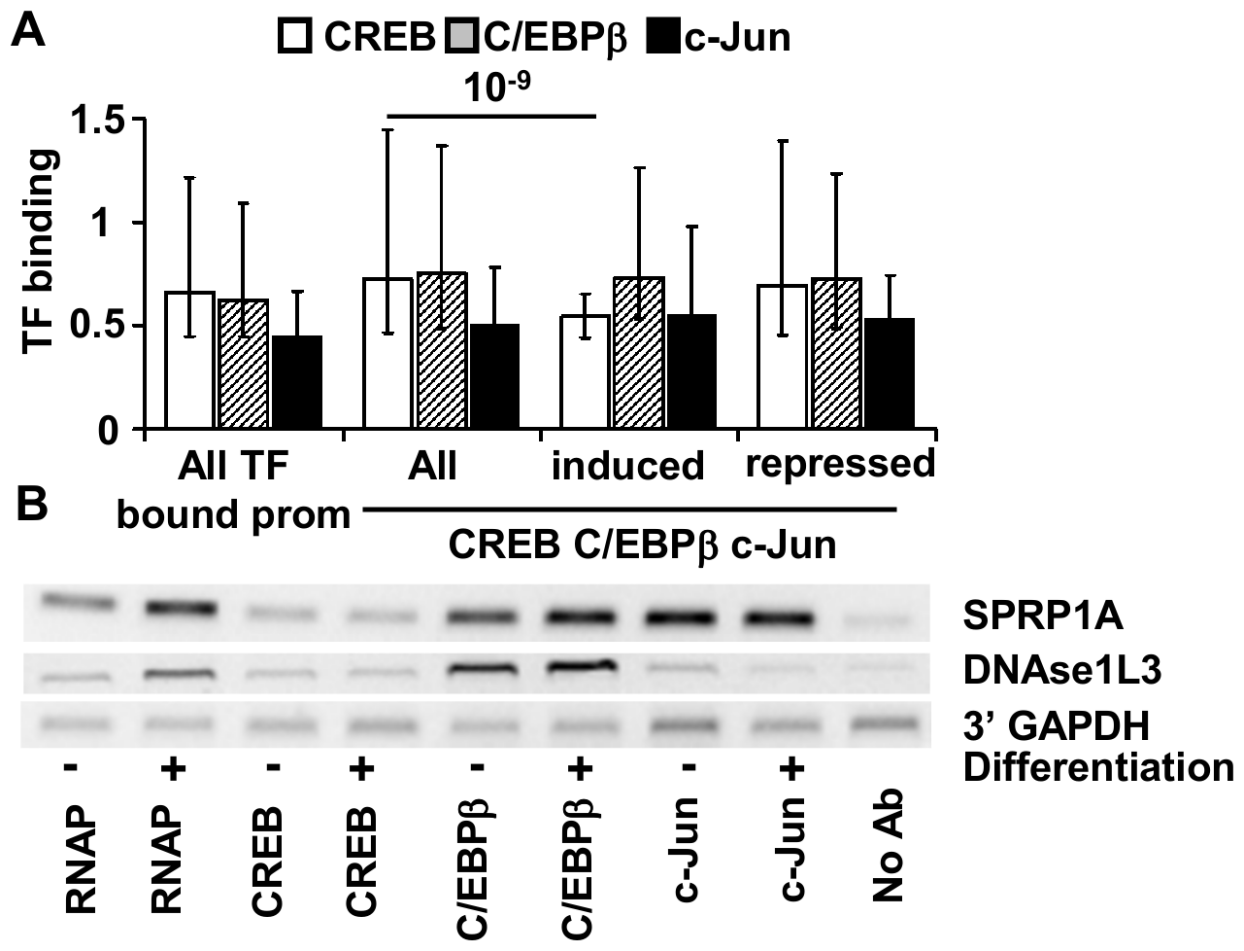
Colocalization of C/EBPβ or c-Jun with CREB determine genes which expression and induction upon differentiation is dependent on C/EBPβ and c-Jun functions. **A.** Fraction of genes repressed by A-C/EBP (left panel) or A-Fos (right panel) in differentiated keratinocytes in groups of promoters bound by different combinations of transcription factors. **B.** Fraction of genes induced by differentiation and repressed by A-C/EBP (left panel) or A-Fos (right panel) in differentiated keratinocytes in groups of genes induced by differentiation which promoters are bound by different combinations of transcription factors. * - numbers are different from expected (p<0.05 using a two-tailed unpaired t-test).

C/EBPβ binding to promoters is induced upon keratinocyte differentiation and RNAP binding is preferentially induced to promoters bound by C/EBPβ and cJun. In order to determine genes that are dependent upon C/EBPβ or cJun function we expressed dominant negatives A-C/EBP and A-Fos using a tetracycline driven expression system [17], [10]. A-C/EBP and A-Fos have been extensively used to study these proteins in a variety of systems [10], [17], [34], [36], [38]. We find that A-C/EBP most strongly represses genes whose promoters are bound by C/EBPβ and not CREB (Figure 2A). Likewise, A-Fos dominant negative preferentially represses genes whose promoters are bound by c-Jun alone or in combination with C/EBPβ (Figure 2A). Because differentiation preferentially induces the expression of genes whose promoters are bound by C/EBPβ and c-Jun, we tested if inhibition of C/EBPβ or cJun would preferentially repress them. We found that A-C/EBP and A-Fos both repress 25% of promoters induced in differentiation, over three times what would be expected by chance, suggesting that differentiation is dependent on these transcription factors. Similar to what is observed for all promoters, A-Fos and A-C/EBP preferentially inhibit genes induced by differentiation if their promoters are bound only by cJun or C/EBPβ (Figure 2B); however the number of induced genes whose promoters are also bound by combinations of transcription factors is relatively low, so many of the differences do not reach statistical significance.

### CREB Binding is Relatively Low for Promoters bound by CREB, C/EBPβ and cJun and Induced by Differentiation

**Figure 3:**
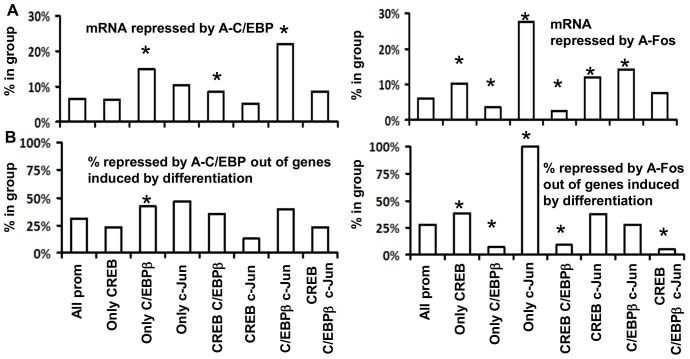
CREB binding is relatively low in the group of promoters bound by CREB, C/EBPβ and cJun and induced by differentiation. **A.** Transcription factor binding distributions in all promoters, promoters bound by CREB, C/EBPβ and c-Jun, and promoters bound by all three of these proteins that are also either induced or repressed upon differentiation. Columns show the mean value of the binding affinity for each of the three transcription factors in these four groups of promoters, while error bars show the 15% and 85% percentiles. The binding affinity of CREB is significantly lower in promoters bound by all three transcription factors and induced by differentiation. Number on top represents the p-value from an unpaired t-test. **B.** Chip-PCR for promoter regions of SPRP1A and DNAse1L3 induced by differentiation. CREB binding is low in comparison with C/EBPβ and c-Jun. 3′ GAPDH region was not enriched in these samples.

Because CREB is rarely bound to promoters induced by differentiation, we hypothesized that CREB binding affinity might be lower in promoters bound by CREB C/EBPβ and cJun and induced by differentiation. Comparison of transcription factor binding distributions in all promoters and promoters bound by CREB, C/EBPβ and cJun in differentiated keratinocytes revealed lower levels of CREB binding in promoters bound by all three of these transcription factors and induced by differentiation (Figure 3A). We arbitrarily selected four genes based on different combinatorial recruitment of transcription factors and induction of RNAP binding upon differentiation and show the ChIP-chip binding patterns for RNAP, CREB, C/EBPβ and c-Jun across their promoters (Figure S4). Claudin4, SPRP1A and DNAse1L3 with no or low CREB binding were induced by differentiation. In contrast, Ribosomal protein 19 with high CREB binding was not. We confirmed binding of transcription factors by Chip-PCR for SPRP1A and DNAse1L3. CREB binding was low in comparison with C/EBPβ and cJun. (Figure 3B).

### Colocalization of C/EBPβ and cJun with CREB is Associated with a High Probability of RNAP Binding in Proliferating or Differentiated Keratinocytes

Colocalization of C/EBPβ and cJun with CREB makes promoters refractory to induction with calcium and to repression by dominant negatives. CREB is known to bind nearly the same set of promoters in different cells [35] suggesting that CREB is involved in housekeeping functions of the cell. These promoters frequently contain CpG islands and typically are highly active. We hypothesized that when C/EBPβ or c-Jun co-localize with CREB, these promoters are strongly bound by RNAP and are not induced by differentiation because other CG binding factors like CREB are already activating them.

**Figure 4:**
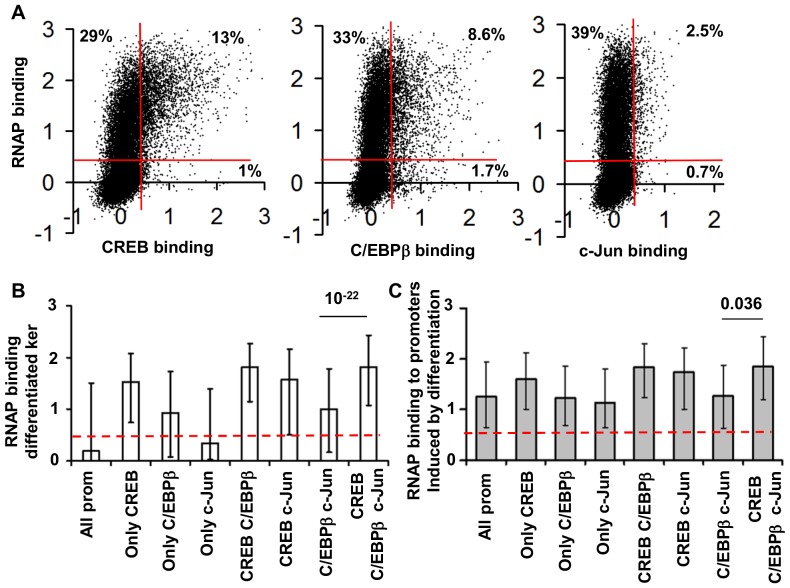
Colocalization of C/EBPβ and c-Jun with CREB is associated with high probability of RNAP binding in all promoters and promoters induced by differentiation. **A.** Scatter plots of transcription factors and RNAP binding show that 95% of promoters bound by CREB, 82% of C/EBPβ and 62% of c-Jun bound promoters are also bound by RNAP. Lines are RNAP binding thresholds. **B.** RNAP binding percentiles (15%, 50% and 85%) in promoters bound by different combinations of transcription factors in differentiated keratinocytes. **C.** RNAP binding percentiles (15%, 50% and 85%) in promoters induced by differentiation and bound by different combinations of transcription factors. Numbers over the bars represent t-test values. Dotted lines are RNAP binding thresholds.

Indeed, scatter plots of transcription factors and RNAP DNA binding (Figure 4A) showed that 93% of promoters bound by CREB, 84% of promoters bound by C/EBPβ and 78% of promoters bound by c-Jun are also bound by RNAP in differentiated keratinocytes (Figure 4A, Figure S5). Analysis of RNAP binding in groups of promoters bound by different combination of transcription factors showed that colocalization of C/EBPβ or c-Jun with CREB corresponds to a high probability of RNAP recruitment (Figure 4 B). 44% of c-Jun only and 72% of C/EBPβ only bound promoters bind RNAP (Table S1). In contrast, when C/EBPβ or c-Jun colocalizes with CREB, about 90% of these promoters bind RNAP (Table S1). Similarly, in undifferentiated keratinocytes colocalization with CREB determines high probability of RNAP recruitment (Figure S5, Table S1). Likewise, when c-Jun colocalizes with C/EBPβ, the distribution of RNAP binding is the same as is observed for promoters bound by C/EBPβ only (Figure 4B). For promoters induced by differentiation we found higher RNAP binding for promoters bound by CREB than bound by C/EBPβ and/or c-Jun (Figure 4C). Despite low CREB binding (Figure 3A), promoters induced by differentiation and bound by CREB, C/EBPβ and c-Jun (Figure 3A) have higher RNAP binding than, promoters induced by differentiation and bound by C/EBPβ and c-Jun only (Figure 4C).

### Gene Promoters that are Methylated and bound by C/EBPβ are Preferentially Induced by Differentiation

**Figure 5:**
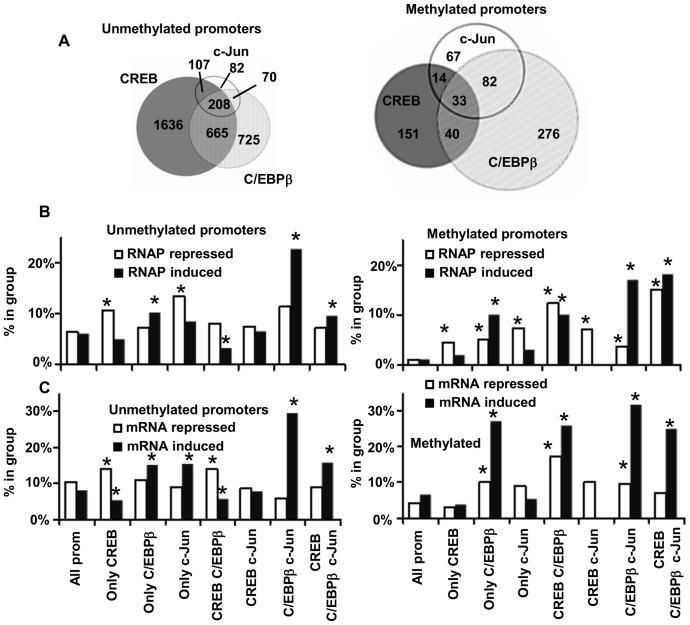
C/EBPβ preferentially binds to methylated promoters and methylated promoters bound by C/EBPβ are preferentially induced by differentiation. **A.** Euler diagrams of methylated and unmethylated promoters bound by different combination of transcription factors show that CREB binding is depleted on methylated promoters while C/EBPβ and c-Jun binding is overrepresented. **B.** Percent of promoters with RNAP is induced or repressed by differentiation in promoters bound by different combinations of transcription factors in differentiated keratinocytes for unmethylated (left) and methylated promoters (right). **C.** Percent of genes with mRNA is induced or repressed by differentiation in promoters bound by different combinations of transcription factors in differentiated keratinocytes for unmethylated (left) and methylated promoters (right). * - numbers are significantly different from expected, (p<0.05).

It was shown that DNA methylation is important for activation of some tissue specific genes involved in keratinocytes differentiation [45]. C/EBPβ and c-Jun can bind methylated CREB binding site, although CREB can not [45]. Thus, the DNA methylation status of the promoters may affect their activation through the combinatorial recruitment of CREB, c-Jun and C/EBPβ. Indeed, C/EBPβ and c-Jun preferentially bind methylated promoters (Figure 5A). Fraction of methylated promoters was 54% for c-Jun and C/EBPβ and less than 14% for promoters where CREB is bound (Table S1).

Similar to what is observed for all promoters, unmethylated promoters containing C/EBPβ and c-Jun are mostly induced by differentiation while those that are also bound by CREB are not (Figure 5B). The fraction of genes for which mRNA is induced by differentiation is higher for methylated in comparison to unmethylated promoters bound by C/EBPβ. 30% of methylated promoters compared to 10% for unmethylated that are bound by both CREB and C/EBPβ are induced by differentiation. (Figures 5B, 5C right panels). Similar to what is observed for promoters induced by differentiation and bound by CREB, c-Jun and C/EBPβ (Figure 3), methylated promoters have relatively low CREB binding (Figure S6). To address functionality of DNA methylation in gene activation upon differentiation, we treated cells with DNA demethylation agent 5-azacytidine (5-AZA) and measured mRNA levels in treated v.s. untreated cells in differentiating keratinocytes. 5-AZA preferentially represses genes whose promoters are bound by C/EBPβ and/or c-Jun but not CREB (Figure S7). Notably, among genes repressed by 5-AZA, bound by C/EBPβ and inhibited by A-C/EBP are markers of keratinocyte differentiation desmocolline and small proline rich protein like 9.

### Consensus and Composite Motifs Containing Two Parts of Consensus Motifs are Enriched in the Groups of Promoters bound by Different Combinations of Transcription Factors

**Figure 6:**
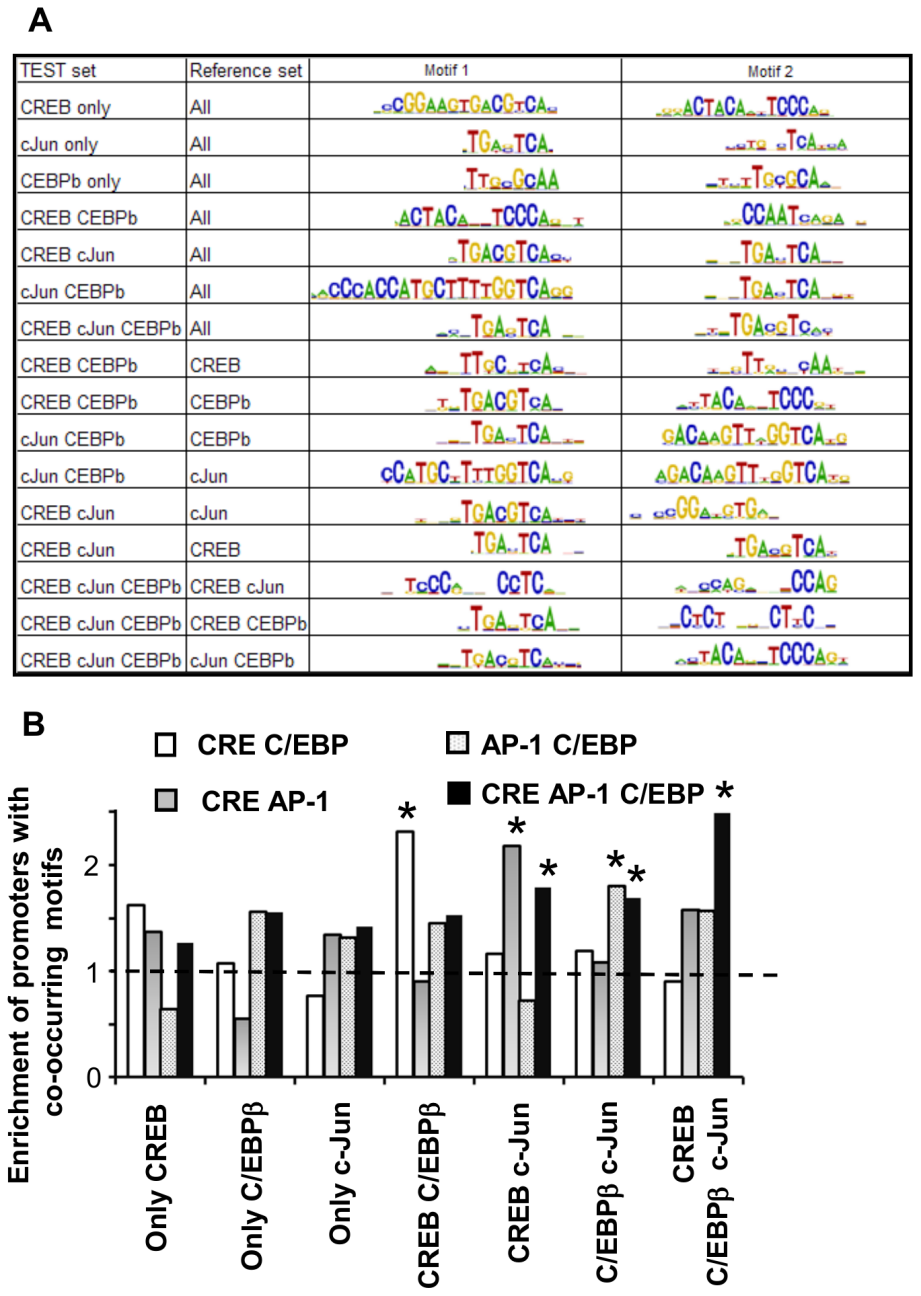
Combinations of consensus motifs and composite motifs are enriched in the groups of promoters bound by different combinations of transcription factors. **A.** Top DNA motifs mostly enriched in test sets v.s. background sets of promoters in the −500 bp…0 bp relative to the transcription start site. Motifs are sorted by enrichment and statistical confidence level using CisFinder. For CREB, c-Jun C/EBPβ set cJun and C/EBPβ bound promoters were used as a background. **B.** Enrichment of promoters containing only two or three transcription factors consensus binding motifs in groups of promoters bound by different combination of transcription factors in undifferentiated keratinocytes. Consensus motifs for CREB (CRE) - TGACGTCA, C/EBPβ - (C/EBP) TTGCGCAA and for c-Jun (AP-1) TGA(C/G)TCA. Note that promoters containing combination of motifs are overrepresented in the groups of promoters bound by corresponding transcription factor. * - numbers are different from expected (p<0.05).

The presence of specific DNA sequences which bind transcription factors is the major mechanism of transcription factor recruitment to the promoters. We hypothesized that specific combinations of DNA binding motifs recruit corresponding combinations of CREB, c-Jun and C/EBPβ. To identify these combinations we calculated motifs overrepresented in sets of promoters bound by transcription factors alone and in different combinations. First, we used all promoters as a background and report the top two enriched motifs in Figure 6A (top seven rows). We found that the most enriched motifs correspond to the known consensus DNA binding sequences. The first DNA binding motif identified for CREB-only was ETS-CREB composite motif, also described in [46]. The second DNA binding motif identified for CREB was ACTACAnnTCCCA and represents a composite ZFP143-RBPJ binding site [47]. For promoters bound by c-Jun and C/EBPβ we found AP1 (TGACTCA) and C/EBPβ (TTGCGCAA ) consensus sequences. For promoters bound by combination of CREB and C/EBPβ we found ACTACAnnTCCCA and CCAAT box. For promoters bound by CREB and c-Jun, CREB (TGACGTCA) and c-Jun (TGAC/G TCA) binding motifs were found. For promoters bound by c-Jun and C/EBPβ we identified new sequence CCCACCATGCTTTGGTCA containing half C/EBP (TTTG) and half c-Jun (GTCA) binding site. And, for promoters bound by all three proteins we found c-Jun and CREB binding sites.

CREB, c-Jun and C/EBPβ can bind promoters via separate binding sites; compete for the same DNA binding motifs or bind DNA as heterodimers. In improve identification of motifs that can recruit two transcription factors, we used as a background promoters bound by one transcription factor (CREB or c-Jun or C/EBPβ, lines 8–13) and calculated motifs enriched in promoters bound by combination of two transcription factors. This analysis identified composite motif containing half C/EBP and half CRE site and C/EBP consensus site for promoters bound by C/EBPβ and CREB relative to CREB. Inversely, when promoters bound by C/EBPβ were used as a background for the same test site we identified CREB binding site and CTACANNTCCC sequence. Likewise, the same calculation performed for the promoters bound by c-Jun and C/EBPβ identified TRE site relative to the C/EBPβ set. Interestingly second identified motif (GACAAGTTAGGTCA) was the same when promoters bound by C/EBP or c-Jun were used as a background. The same motif was identified for c-Jun and C/EBPβ bound promoters relatively to c-Jun. Motifs enriched in CREB –c-Jun bound promoters relative to c-Jun or CREB represent known binding motifs for corresponding transcription factors. The same calculations performed for combination of three transcription factors identified consensus motifs for c-Jun and CREB and TCCCANNNCCTC for C/EBPβ.

### Consensus DNA Binding Motifs are Preferentially Colocalize in Promoters bound by Combinations of Corresponding Transcription Factors

Data in Figure 6A suggest that consensus and combination of consensus motifs for transcription factors are enriched in the groups of promoters bound by combination of corresponding transcription factors. We asked if consensus motifs co-occur in the same promoters. Consensus motifs for CREB (CRE) TGACGTCA, C/EBPβ TTGCGCAA and c-Jun (TRE) TGA(C/G)TCA were used. We found that promoters containing combinations of consensus DNA binding motifs are overrepresented in the promoters bound by corresponding combinations of transcription factors (Figure 6B).

This analysis suggests that combinatorial sited and combination of consensus transcription factor binding sites lead to specific combinatorial recruitment of transcription factors and determine functionality of promoters.

## Discussion

Our results suggest that during keratinocyte differentiation, the activation of gene expression is regulated by promoter-specific combinations of CREB, c-Jun and C/EBPβ. Specifically, C/EBPβ binding is induced upon differentiation and promoters bound by C/EBPβ, either alone or in combination with c-Jun with no or low CREB binding are preferentially induced by differentiation. Colocalization of c-Jun and C/EBPβ with CREB on the promoter corresponds to a high probability of RNAP recruitment in differentiated or proliferating keratinocytes. Moreover, relatively large fraction of genes where CREB is bound to promoters alone or in combination with C/EBPβ has higher expression in proliferating compared to differentiated keratinocytes.

Dominant negatives A-C/EBP and A-Fos inhibited expression of genes whose promoters are not bound by CREB. The role of C/EBPs and AP-1 in keratinocytes differentiation and proliferation is well established [2], [5]–[8], [22], [25]. Our data confirmed our initial hypothesis that C/EBPβ and c-Jun regulate keratinocyte differentiation when they do not colocalize with CREB. The fact that promoters bound by combination of C/EBPβ and c-Jun are preferentially induced by differentiation is new and was not expected. This data also suggest that CREB works as a dominant transcription factor both in inducing gene expression and preventing it from being affected by other transcription factors. The existence of small group of promoters that are bound by CREB but not RNAP suggests that some regulatory mechanisms prevent them from being activated. CREB is known to bind the same set of promoters in different cell types suggesting that it is involved in housekeeepig cell functions [35]. It is also known to mediate IL-1 induced Fos expression in keratinocytes [48], stimulate transcription of gluconeogenic genes in liver [49], involved in memory formation [50] as well as other cell type specific functions. Concordantly, our data shows that promoters bound by CREB, c-Jun and C/EBPβ are preferentially induced upon differentiation. Thus, it is the combinatorial recruitment of transcription factors that determines whether a gene is going to be repressed, activated or experience no change in response to specific stimulus.

Our data identified only part of combinatorial logic which regulates genes expression during keratinocytes differentiation. Existence of promoters bound by the same combination of transcription factors and either induced or repressed by differentiation suggest that other factors (besides CREB identified in this study) bound to promoters together with C/EBPβ and c-Jun will determine if specific gene is ultimately induced, repressed or do not change upon differentiation. Many other transcription factor are involved in keratinocytes differentiation [1], [2], [3], [4] and, it would be interesting to identify whether they are functioning on the same promoters cooperatively activated by C/EBPβ and c-Jun.

Notably, analysis of combinatorial recruitment of CREB, c Jun and C/EBPα generated similar results (Figure S8 and Table S1).

Presence of specific DNA sequences in the promoter determines recruitment of corresponding transcription factors for regulation of gene expression. CREB, c-Jun and C/EBPβ can compete for the same binding sites or bind simultaneously at different sequences. c-Jun, C/EBPβ and CREB bind to TGACTCA, TTGCGCAA and TGACGTCA consensus sequences respectively. CREB and c-Jun can bind the same TGACGTCA [13], [35], [51] sequence and C/EBPα - c-Jun heterodimer binds TTGCGTCAT sequence [30], whose core element, CGTCA, also can be bound by CREB [28], [29], [35]. DNA methylation can regulate differential binding of transcription factors. For example, methylation of CRE inhibits binding of CREB but promotes binding of C/EBPβ and C/EBPα and does not influence c-Jun binding [45]. Data presented in this paper suggest that combinatorial recruitment of transcription factors induces activation of genes during differentiation in a different manner for methylated compared to unmethylated promoters. CREB binding to methylated promoters bound by C/EBPβ was low and these promoters were more often induced by differentiation than unmethylated promoters bound by C/EBPβ and CREB.

To understand how sequences of promoters determine preferential recruitment of CREB, c-Jun and C/EBPβ in different combinations, we identified DNA motifs overrepresented in promoters bound by one, two or all three of these transcription factors relative to all promoters or relative to promoters bound by one or two transcription factors. As expected, we found that consensus binding sites are overrepresented in promoters bound by single transcription factor or in groups where corresponding transcription factors co-localize with other transcription factors. As expected, when promoters bound by a particular transcription factor were used as a background, subsets of promoters bound both by this and another transcription factor where enriched in the other transcription factor’s consensus sequence. The composite motif between CREB and C/EBPβ binding motif identified in our analysis was similar to the one reported in [28], [29]. Several studies suggest that CREB and C/EBPβ do not interact but compete for the same sequence [28], [26]. Interestingly, novel long motif CCCACCATGCTTTTGGTCA is identified in promoters bound by C/EBPβ and c-Jun. Similar composite C/EBPβ - c-Jun motif GACAAGTT(T/A)GGTCA is enriched in promoters bound by C/EBPβ and c-Jun relatively either to c-Jun or C/EBPβ bound promoters. This suggests that it is the C/EBPβ/c-Jun protein complex that binds to this motif, similar to what is described in papers [30], [31].

This paper uncovered combinatorial rules of transcription factor recruitment that determine activation of gene expression upon differentiation.

## Methods

### Ethics Statement

All mouse experiments were approved by the Animal Ethics Committee at the National Institutes of Health, under the approved protocol number LM-076. Newborn mice were handled and humanely sacrificed by carboxyl dioxide followed by secondary physical method in accordance with the National Institutes of Health Institutional Guidelines (NCI, NIH, Bethesda, MD, USA).

### Primary Keratinocytes Cultures

Keratinocytes were cultured as describe in [52] from new-born wild type FVB mice or from mice expressing A-C/EBP or A-Fos dominant negatives of C/EBP’s or AP-1 in keratinocytes under the control of tetracycline inducible repressor [10], [17]. Primary keratinocytes were seeded at a density of one mouse epidermis per 10 cm dish or equivalent in calcium and magnesium free S-MEM (GIBCO Laboratories, Grand Island, N.Y), supplemented with 8% Chelex (Bio-Rad, Richmond, CA) treated FBS (Atlanta Biologicals, Inc) and 0.2 mM calcium (CaCl_2_). After 24 hours, cultures were washed twice with PBS and switched to medium with 0.05 mM calcium (low calcium). At the same time doxycyclin added to the media of A-C/EBP or A-Fos cultures was removed to induce expression of A-C/EBP or A-Fos. 24 hours later, cultures were switched to the same medium with 0.4 mM calcium (Hi calcium) for two days to induce differentiation or maintained in parallel as undifferentiated keratinocytes in the low calcium medium. For demethylation experiments, 5-azacytidine (5-aza) was added 6 hr after seeding to the culture medium to a final concentration of 1 µM. Cell culture medium was replaced every day.

### Chromatin Immunoprecipitation (ChIP)

ChIP experiments were performed following the protocol from Farnham’s group (http://farnham.genomecenter.ucdavis.edu/protocol.html). Primary cultured cells were chemically cross-linked by adding 0.6% formaldehyde (Sigma) directly to the medium. Cells were allowed to cross-link for 10 minutes with gentle swirling at room temperature. The cross-linking reaction was stopped by adding 125 mM glycine and dishes were swirled for 5 minutes at room temperature. Cells were washed twice with ice-cold PBS and harvested in ice-cold PBS containing protease (Complete mini, Roche) and phosphatase inhibitors (1 mM NaF, 1 mM Na_3_VO_4_). Cells were pelleted by centrifugation at 4°C for 5 minutes at 300 g. Cells where incubates in cell lysis buffer (5 mM PIPES pH 8.0 85 mM KCL 0.5% NP40 1 mM NF 1 mM NaVa Roche protease inhibitors cocktail) and resuspended in nuclear lysis buffer (50 mM Tris-Cl pH 8.1 10 mM EDTA, 1% SDS proteases and phosphates inhibitors as above) and sonicated to average size of 500 bp. After centrifugation supernatant was diluted 3 times by dilution buffer (0.01% SDS, 1.1% Trition×100, 1.2 mM EDTA, 16.7 mM Tris-Cl pH 8.1, 167 mM NaCl). 300 l of sonicated chromatin preparation with protein concentration 1–2 µg/µl (determined using BCA, PIERCE) was incubated overnight with antibodies. ChIP was performed using antibodies against RNA pol II (RNAP) (20 µg/ml final, Covance, 8WG16) that recognizes the unphosphorylated form of RNAP, CREB (2 µg/ml of the each antibody from Santa Cruz (sc-186) and Upstate (06–863)), H3K9 acetyl from Upstate 06–942, c-Jun from Santa Cruz (10 ug/ml, sc-1694) and C/EBPβ from Santa Cruz (10 µg/ml, sc-150). A fraction of lysate was left untreated to serve as an input control. Immunocomplexes were captured using protein G agarose beads (Invitrogen) blocked with 1 g/l yeast tRNA and BSA (Sigma) and washed twice with the buffer containing 2 mM EDTA, 100 mM Tris-Cl pH 8.0 and 0.18% Sarkosyl, and four times with the Chip wash buffer (100 mM Tris-Cl pH 8.5, 500 mM LiCl, 1% NP40, 1% deoxycholic acid) and two times with TE. After incubating with RNAse A and Proteinase K, DNA was eluted using QIAquick PCR Purification Kit. From 1–2×10^6^ primary keratinocytes for RNAP, CREB, and C/EBPβ, 0.5–2 ng of DNA were typically immunoprecipitated. DNA quantification was performed using Picogreen DNA quantification kit (Invitrogen, USA). PCR was performed using RedMix Tag polymerase (Sigma).

### ChIP DNA Amplification and Hybridization

Primers conjugated with Cy3 or Cy5 (Sigma Genosys, USA) were used for the amplification of the input or immunoprecipitated DNA using round A/B/C random amplification protocol: http://research.stowers-institute.org/gertonlab/protocols/RandomPCRamplification.pdf. Only one round of amplification was used. After amplification 5–6 µg of ChIP or control DNA was purified using PCR Purification Kit (Qiagen), isopropanol precipitated, and vacuum dried for 5 minutes. DNA was dissolved in 3 µl water, mixed with Component A and Hybridization buffer (Nimblegen) according to manufacturer’s instructions. Amplified ChIP DNA was hybridized overnight to Nimblegen MM5 min Mouse promoter microarrays containing 400,000 oligos interrogating 26,264 promoters in MAUI hybridization station (BioMicro Systems) and washed according to manufacturer instruction. Arrays were dried by centrifugation and scanned using Axon 4000 B scanner. Images were processed with NimbleScan (Nimblegen) using default settings.

### Calculation of Transcription Factors DNA Binding

Nimblegen MM5 min promoter arrays contain probes for 26,264 promoter regions spanning approximately −1000 bp…+500 bp relative to the transcription start site. We limited our data analysis to 20,328 promoters. We excluded promoters that are located on the X or Y chromosomes, 3,940 promoters were further eliminated that either had unsequenced DNA segments larger than 150 bp or which contained large regions of DNA sequence (≥150 bp) which were identical to regions in 10 or more of other promoters. Binding of given transcription factor was defined as an average enrichment of ChIP DNA over input DNA for the whole −1000…+500 promoter region. Binding from individual replicates were averaged. Promoters were defined as bound by several transcription factors if binding of these factors were higher than specific thresholds. Values of thresholds for calculation of transcription factor DNA binding in Ln2: CREB - 0.4, C/EBPβ-0.4, C/EBPα-0.36 c-Jun-0.36, RNAP-0.4. RNAP induction or repression upon differentiation: RNAP Diff>0.4 and RNAP Diff- RNAP Undiff>0.3. H3K9 acetylation induction or repression upon differentiation: H3K9Ac Diff>0.4 and H3K9Ac Diff- H3K9Ac Undiff>0.36.

### Analysis of DNA Methylation

MeDIP experiments were described in Rishi [45].

### Affymetrix Gene Expression Profiling

mRNA expression profiling with Affymetrix microarrays (Mouse genome 430 2.0 array) was performed by NCI microarray core facility (Frederick). We compared the mRNA expression levels of genes determined using mRNA expression arrays to the binding of proteins to the promoters regions of those genes, determined by ChIP-chip data collected using Nimblegen promoter chips. Using GenBank Accessions, Gene Symbols, UniGene Clusters and UniGene IDs the mRNA expression data was mapped to the same promoter set as the ChIP-chip data. Genes in the expression data which shared a common identifier with promoters in the ChIP-chip data were assigned to their matching promoters in the ChIP-chip set. When multiple data points from the mRNA expression data were all mapped to the same promoter in the ChIP-chip data, the average of these points was assigned to that promoter. 17,930 of the 20,328 promoters used in our ChIP-chip analysis were successfully assigned mRNA expression values. Threshold for mRNA changes upon differentiation – more than 1.4 or 0.5 in Ln2 scale.

### DNA Motif Analysis

DNA Motif analysis was performed using CisFinder http://lgsun.grc.nia.nih.gov/CisFinder/ [53]. For calculation of motifs colocalization, number of motifs colocalizing in groups of promoters bound by different combinations of CREB, C/EBPβ and c-Jun was normalized to number of colocalizing motifs in all promoters.

### Statistical Analysis

Excel “Chitest” function was used to calculate significance of observed values from expected ones based on the total number of events. Excel two tailed “Ttest” function was used to determine whether two samples are likely to have come from the same two underlying populations that have the same mean.

### GEO Accession Number

GSE48383.

## Supporting Information

Figure S1 **Promoters induced by differentiation are overrepresented in the group of C/EBPβ bound and promoters with increase of C/EBPβ binding upon differentiation. A.** Scatterplotts of transcription factors and RNAP before and after differentiation show that binding do not change for majority of promoters with the highest scatter for RNAP and overall increase of C/EBPβ binding. **B.** Scatterplotts of changes in RNAP v.s. transcription factors upon differentiation show that promoters induced by differentiation are overrepresented in group of promoters with induced C/EBPβ binding. **C.** Scatterplotts of changes in RNAP upon differentiation v.s. binding of transcription factors in differentiated keratinocytes show that promoters induced by differentiation are overrepresented in group of promoters bound by C/EBPβ. **D.** Efficiency of c-Jun immunoprecipitation was about 100%: 5% of input cell lyzat and 20% of c-Jun Chip material was resolved by SDS-PAGE transferred to membrane and probed with c-Jun antibody.
(TIF)

Figure S2 **Colocalization of c-Jun and C/EBPβ with CREB determine levels of mRNA induction upon differentiation. A.** Inductionof RNAP binding (15%, 50%, 85% percentiles) in differentiated compared to undifferentiated keratinocytes for promoters where RNAP is induced by differentiation and bound by different combination of transcription factors. **B.** Increase of mRNA levels (15%, 50%, 85% percentiles) in differentiated compared to undifferentiated keratinocytes for genes whose mRNA is induced by differentiation and promoters are bound by different combination of transcription factors. Numbers represent t-test values. Dotted lines represent thresholds for induction.
(TIF)

Figure S3 **H3K9 acetylation is preferentially induced by differentiation when promoters are bound by combination of C/EBPβ and c-Jun. A.** Scatterplot of RNAP binding versus H3K9 acetylation in differentiated keratinocytes. **B.** Changes of RNAP binding upon differentiation correlates with changes in H3K9 acetylation. **C.** Fraction of promoters bound by different combinations of transcription factors in differentiated keratinocytes where H3K9 acetylation is reduced (white bars) or induced upon differentiation (black bars). * - numbers are different from expected (p<0.05). **D.** Induction of H3K9 acetylation (15%, 50%, 85% percentiles) for promoters where H3K9 acetylation is induced and bound by different combinations of transcription factors in differentiated keratinocytes. Numbers represent t-test values. Dotted line represents threshold for induction.
(TIF)

Figure S4 **Examples of binding patterns of RNAP, CREB, C/EBPβ and c-Jun across promoter regions of selected promoters that are induced or not induced by differentiation.** Claudin4 promoter induced by differentiation, not bound by CREB and bound by C/EBPβ and c-Jun, Small proline rich protein 1A promoter induced by differentiation, not bound by CREB (CREB average binding 0.36, just under threshold 0.4) and bound by C/EBPβ and c-Jun, DNAse1-like3 induced by differentiation and bound by C/EBPβ, c-Jun with low CREB binding, Rsp19 - ribosomal protein 19 promoter not induced by differentiation, bound by C/EBPβ and c-Jun with strong CREB binding. Arrows on top are directions of transcription started from transcriptional start site.
(TIF)

Figure S5 **Colocalization of C/EBPβ and c-Jun with CREB is associated with high probability of RNAP binding in undifferentiated keratinocytes.** RNAP binding percentiles (15%, 50% and 85%) for promoters bound by different combinations of transcription factors in undifferentiated keratinocytes.
(TIF)

Figure S6 **CREB binding is relatively low when promoters are methylated while C/EBPβ biding to methylated or unmethylated promoters is the same.** Promoters bound by different combination of transcription factors are shown.
(TIF)

Figure S7 **Genes with promoters bound by C/EBPβ and c-Jun are preferentially repressed by 5-azacytozine.** Percent of genes which mRNA is repressed (white bars) or induced (black bars) by DNA demethylation agent 5-azacytidine in groups of promoters bound by different combinations of transcription factors in differentiated keratinocytes. * - significant difference p<0.05.
(TIF)

Figure S8 **Combinatorial recruitment of CREB, C/EBPα and c-Jun determines activation of promoters in keratinocyte differentiation. A.** Scatterplot of C/EBPα binding v.s. C/EBPβ binding show that C/EBOa was detected on a subset of promoters bound by C/EBPβ. **B.** RNAP binding percentiles (15%, 50% and 85%) in promoters bound by different combinations of CREB, C/EBPα and c-Jun. **C.** Fraction of promoters bound by different combination of transcription factors in differentiated keratinocytes where RNAP binding is repressed (white bars) or induced by differentiation (black bars). **D.** Fraction of genes with mRNA levels induced or repressed by differentiation more than 1.4 times in groups of promoters bound by different combinations of transcription factors. * - values are different from expected (p<0.05). ). **E.** Fraction of genes repressed by A-C/EBP differentiated keratinocytes in groups of promoters bound by different combinations of transcription factors. **F.** Fraction of genes repressed by A-Fos in differentiated keratinocytes in groups of promoters bound by different combinations of transcription factors * - numbers are different from expected (p<0.001).
(TIF)

Table S1 **Numbers and fractions of promoters bound by different combinations of transcription factors and induced or repressed by differentiation.** Reference groups used to calculate fractions in different groups are also presented. Above row 64 is the data for C/EBPβ, CREB and c-Jun and below row 67 is the data for C/EBPα, CREB and c-Jun.
(XLS)
