# Correction: Combinatorial Recruitment of CREB, C/EBPβ and c-Jun Determines Activation of Promoters upon Keratinocyte Differentiation

**DOI:** 10.1371/annotation/0cd737c6-949b-48f9-8bc8-ab36376c03e3

**Published:** 2013-12-31

**Authors:** Julian M. Rozenberg, Paramita Bhattacharya, Raghunath Chatterjee, Kimberly Glass, Charles Vinson

Figures 2 and 3 were incorrectly switched. The image currently appearing as Figure 2 belongs with the title and legend for Figure 3, and the image currently appearing as Figure 3 belongs with the title and legend for Figure 2. The titles and legends themselves are in correct order.

