# Supplementary figures and images for: Combinatorial Recruitment of CREB, C/EBPβ and c-Jun Determines Activation of Promoters upon Keratinocyte Differentiation
Source: PLoS One. 2013 Nov 7;8(11):e78179. doi: 10.1371/journal.pone.0078179 (PMC3820678; doi:10.1371/journal.pone.0078179)

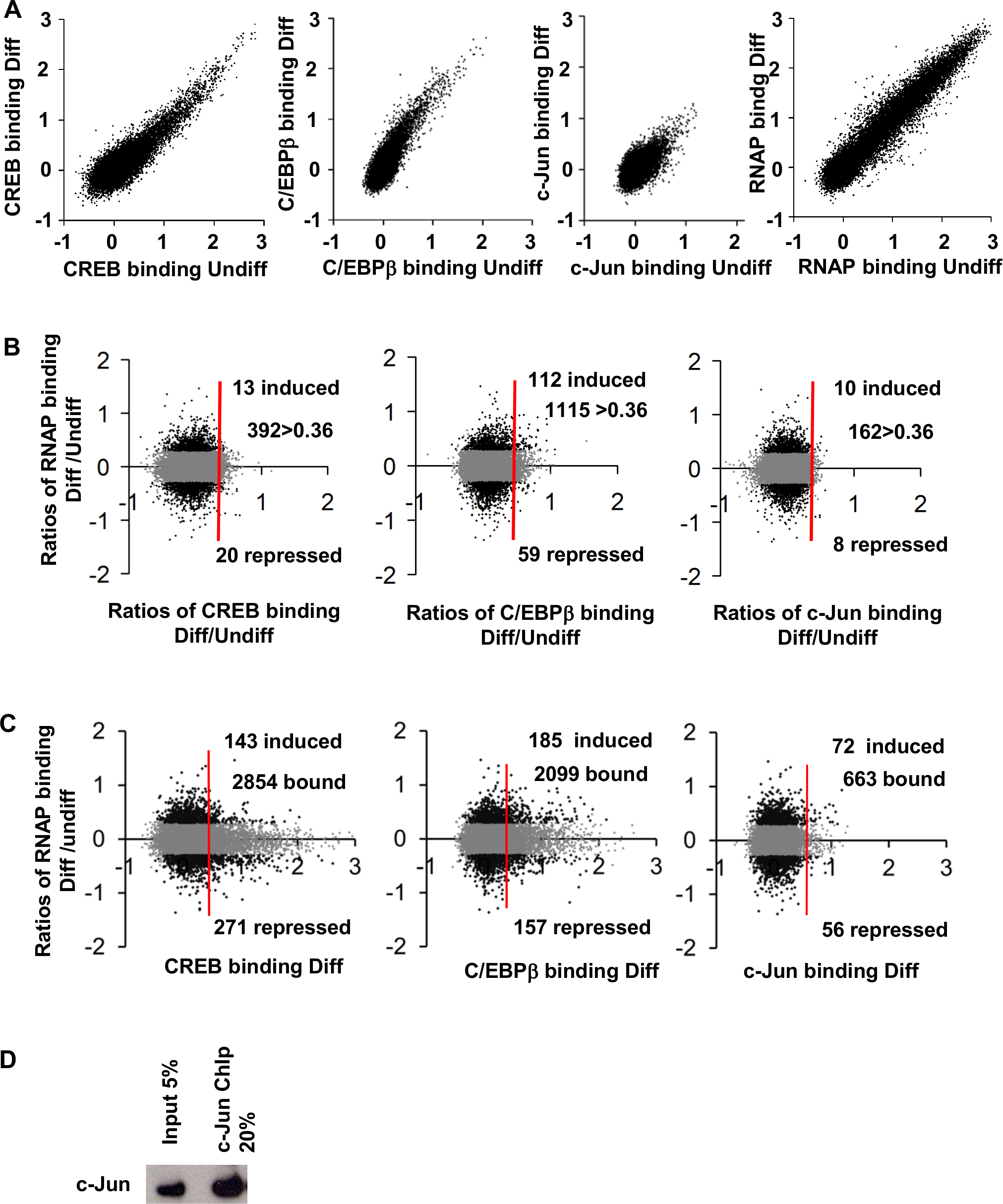

Supplement: Figure S1 — Promoters induced by differentiation are overrepresented in the group of C/EBPβ bound and promoters with increase of C/EBPβ binding upon differentiation. A. Scatterplotts of transcription factors and RNAP before and after differentiation show that binding do not change for majority of promoters with the highest scatter for RNAP and overall increase of C/EBPβ binding. B. Scatterplotts of changes in RNAP v.s. transcription factors upon differentiation show that promoters induced by differentiation are overrepresented in group of promoters with induced C/EBPβ binding. C. Scatterplotts of changes in RNAP upon differentiation v.s. binding of transcription factors in differentiated keratinocytes show that promoters induced by differentiation are overrepresented in group of promoters bound by C/EBPβ. D. Efficiency of c-Jun immunoprecipitation was about 100%: 5% of input cell lyzat and 20% of c-Jun Chip material was resolved by SDS-PAGE transferred to membrane and probed with c-Jun antibody. (TIF) [file pone.0078179.s001.tif]

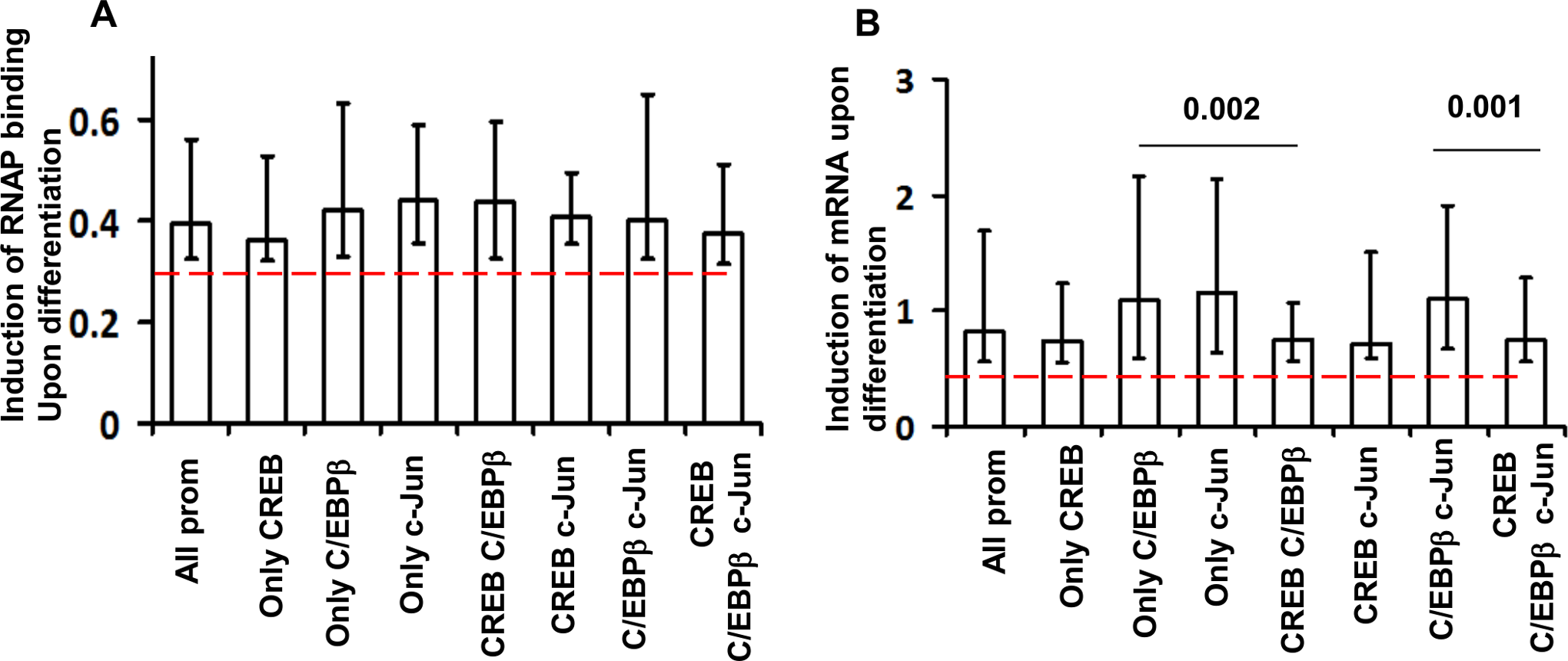

Supplement: Figure S2 — Colocalization of c-Jun and C/EBPβ with CREB determine levels of mRNA induction upon differentiation. A. Inductionof RNAP binding (15%, 50%, 85% percentiles) in differentiated compared to undifferentiated keratinocytes for promoters where RNAP is induced by differentiation and bound by different combination of transcription factors. B. Increase of mRNA levels (15%, 50%, 85% percentiles) in differentiated compared to undifferentiated keratinocytes for genes whose mRNA is induced by differentiation and promoters are bound by different combination of transcription factors. Numbers represent t-test values. Dotted lines represent thresholds for induction. (TIF) [file pone.0078179.s002.tif]

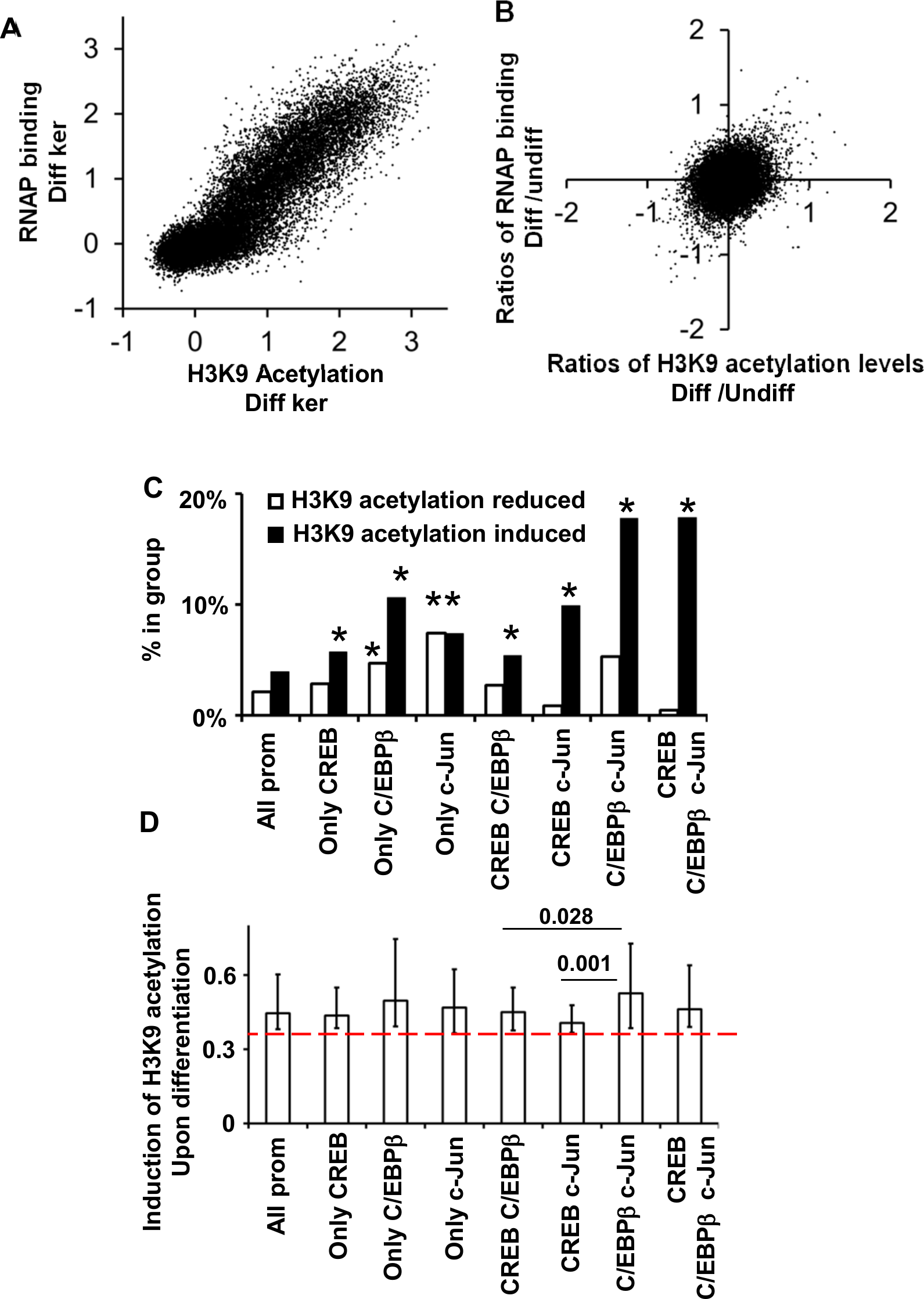

Supplement: Figure S3 — H3K9 acetylation is preferentially induced by differentiation when promoters are bound by combination of C/EBPβ and c-Jun. A. Scatterplot of RNAP binding versus H3K9 acetylation in differentiated keratinocytes. B. Changes of RNAP binding upon differentiation correlates with changes in H3K9 acetylation. C. Fraction of promoters bound by different combinations of transcription factors in differentiated keratinocytes where H3K9 acetylation is reduced (white bars) or induced upon differentiation (black bars). * - numbers are different from expected (p<0.05). D. Induction of H3K9 acetylation (15%, 50%, 85% percentiles) for promoters where H3K9 acetylation is induced and bound by different combinations of transcription factors in differentiated keratinocytes. Numbers represent t-test values. Dotted line represents threshold for induction. (TIF) [file pone.0078179.s003.tif]

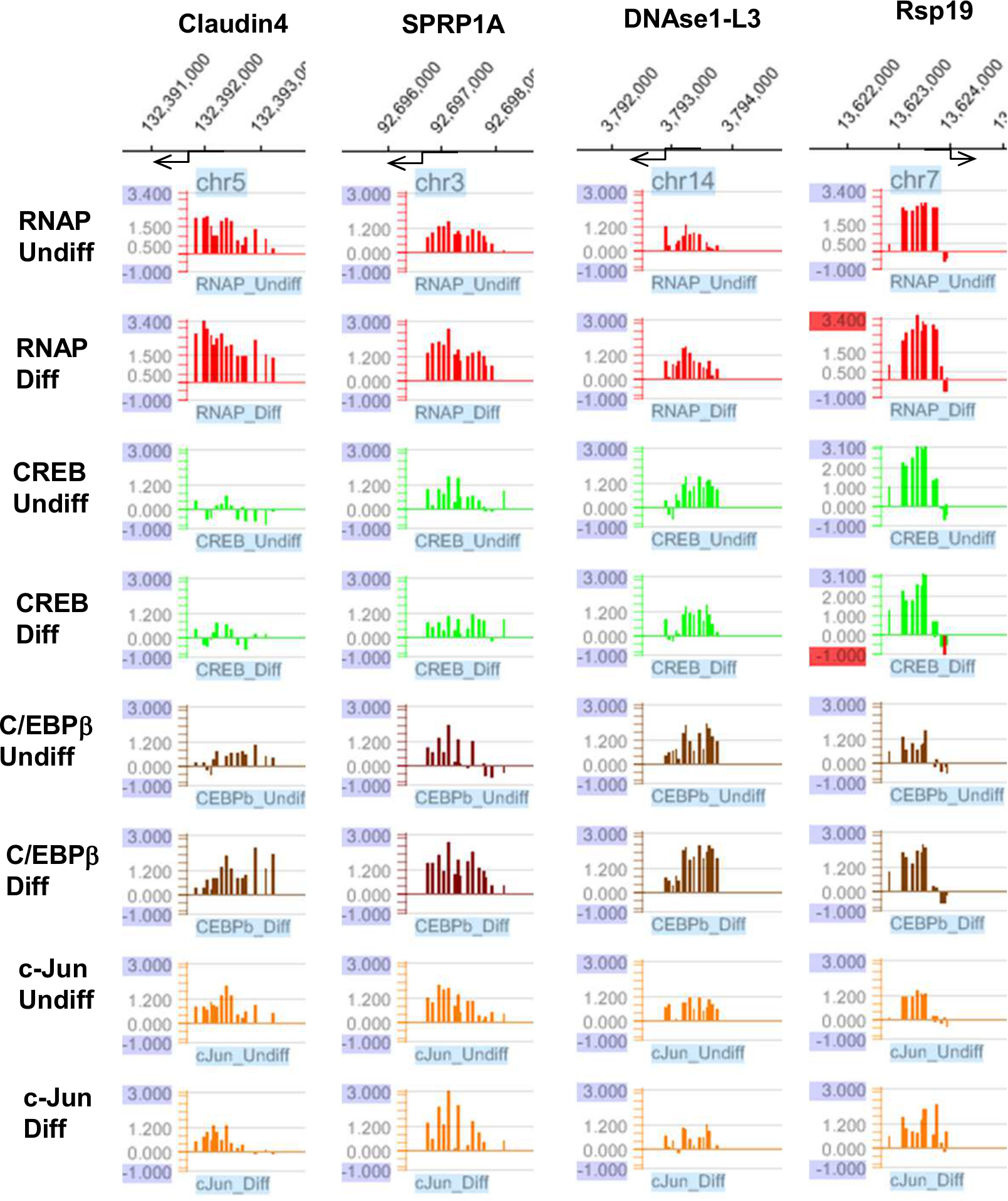

Supplement: Figure S4 — Examples of binding patterns of RNAP, CREB, C/EBPβ and c-Jun across promoter regions of selected promoters that are induced or not induced by differentiation. Claudin4 promoter induced by differentiation, not bound by CREB and bound by C/EBPβ and c-Jun, Small proline rich protein 1A promoter induced by differentiation, not bound by CREB (CREB average binding 0.36, just under threshold 0.4) and bound by C/EBPβ and c-Jun, DNAse1-like3 induced by differentiation and bound by C/EBPβ, c-Jun with low CREB binding, Rsp19 - ribosomal protein 19 promoter not induced by differentiation, bound by C/EBPβ and c-Jun with strong CREB binding. Arrows on top are directions of transcription started from transcriptional start site. (TIF) [file pone.0078179.s004.tif]

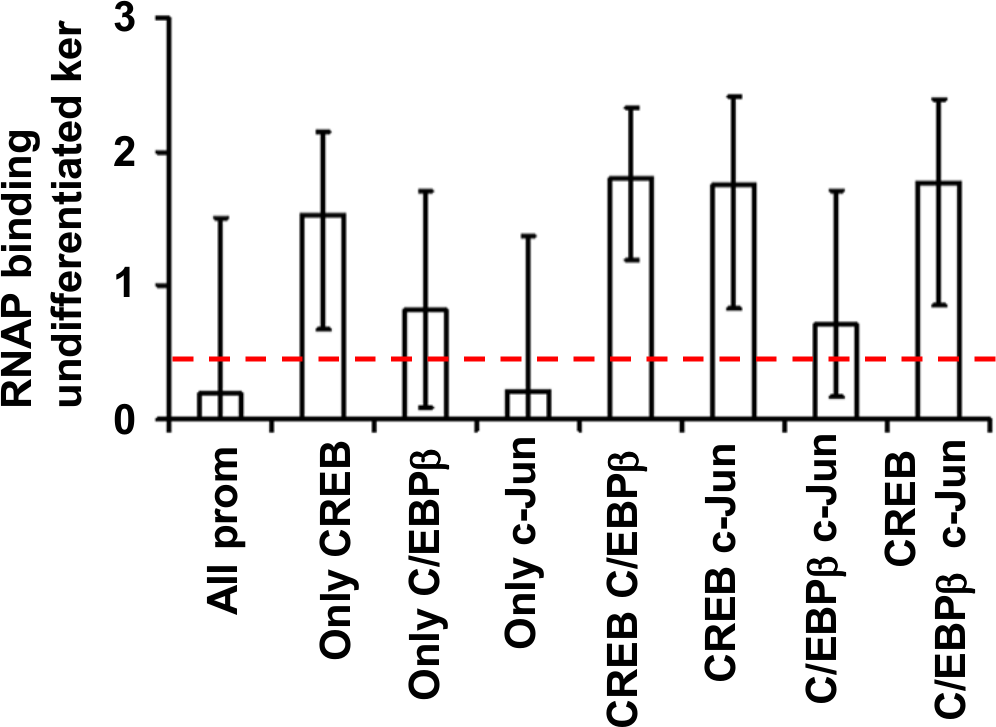

Supplement: Figure S5 — Colocalization of C/EBPβ and c-Jun with CREB is associated with high probability of RNAP binding in undifferentiated keratinocytes. RNAP binding percentiles (15%, 50% and 85%) for promoters bound by different combinations of transcription factors in undifferentiated keratinocytes. (TIF) [file pone.0078179.s005.tif]

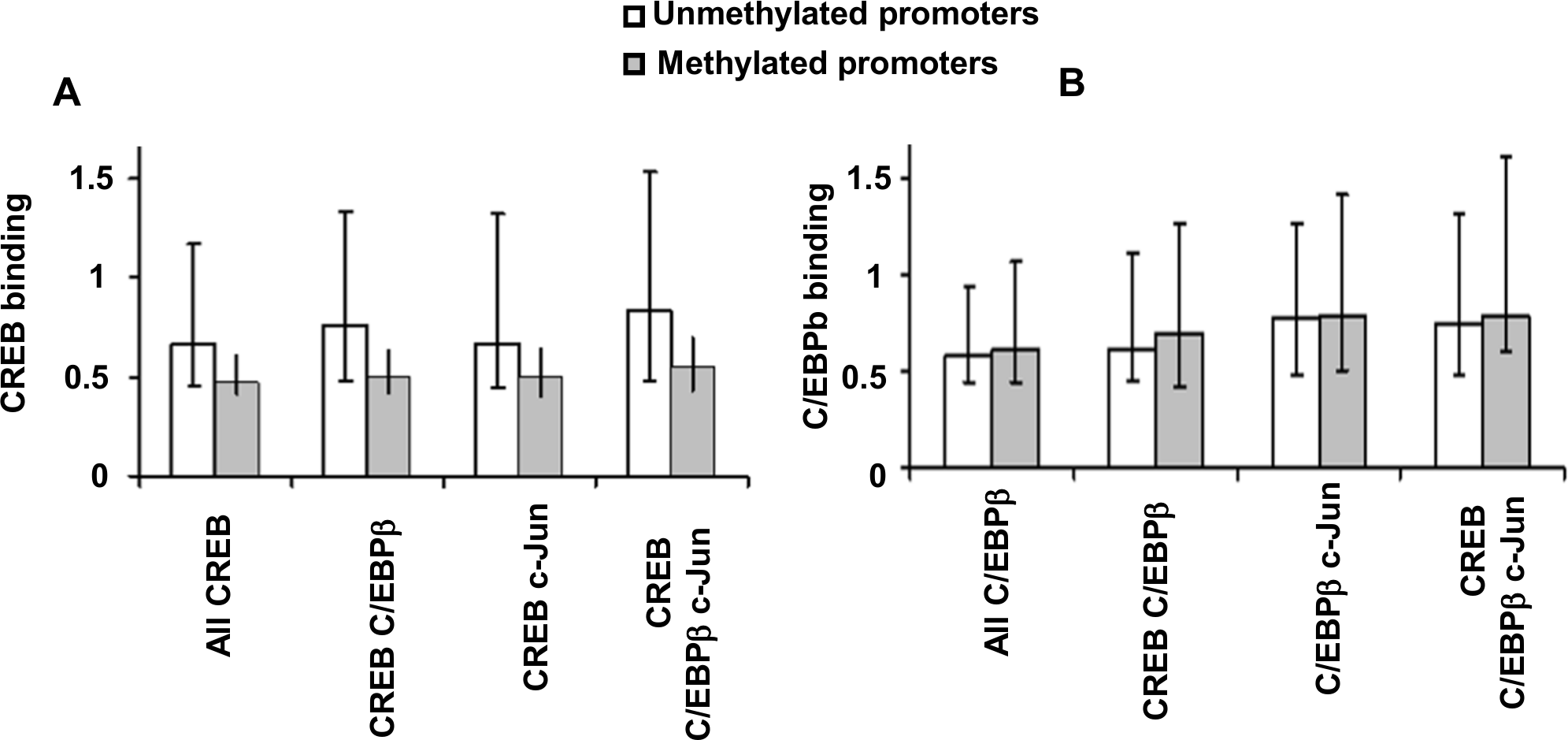

Supplement: Figure S6 — CREB binding is relatively low when promoters are methylated while C/EBPβ biding to methylated or unmethylated promoters is the same. Promoters bound by different combination of transcription factors are shown. (TIF) [file pone.0078179.s006.tif]

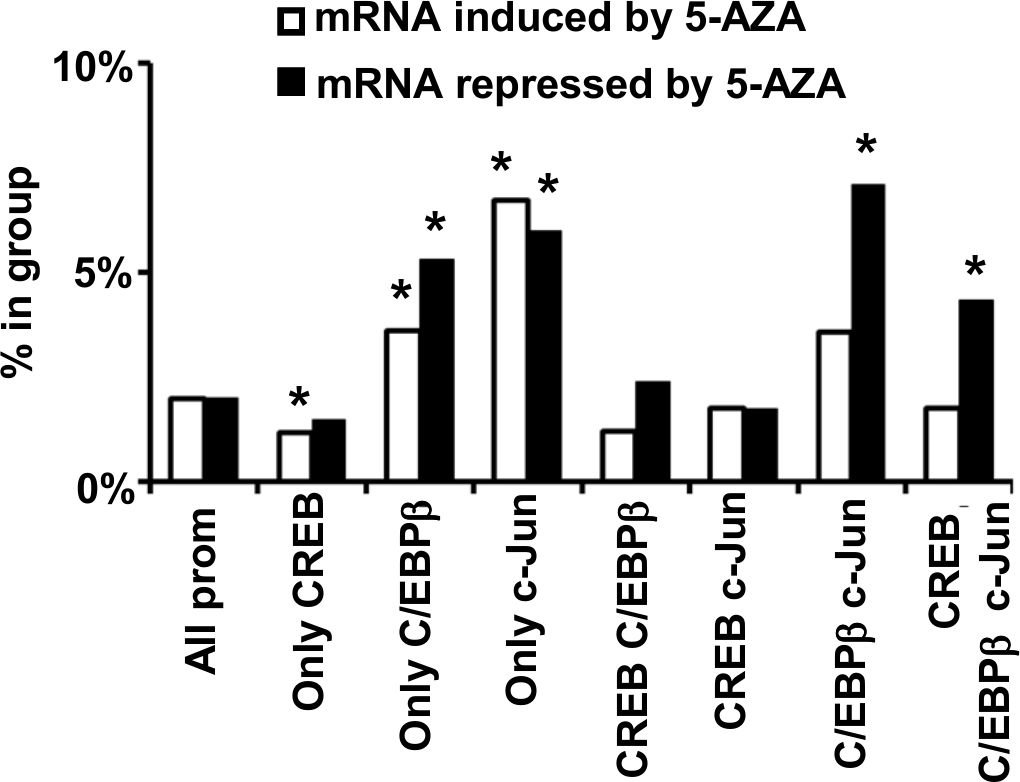

Supplement: Figure S7 — Genes with promoters bound by C/EBPβ and c-Jun are preferentially repressed by 5-azacytozine. Percent of genes which mRNA is repressed (white bars) or induced (black bars) by DNA demethylation agent 5-azacytidine in groups of promoters bound by different combinations of transcription factors in differentiated keratinocytes. * - significant difference p<0.05. (TIF) [file pone.0078179.s007.tif]

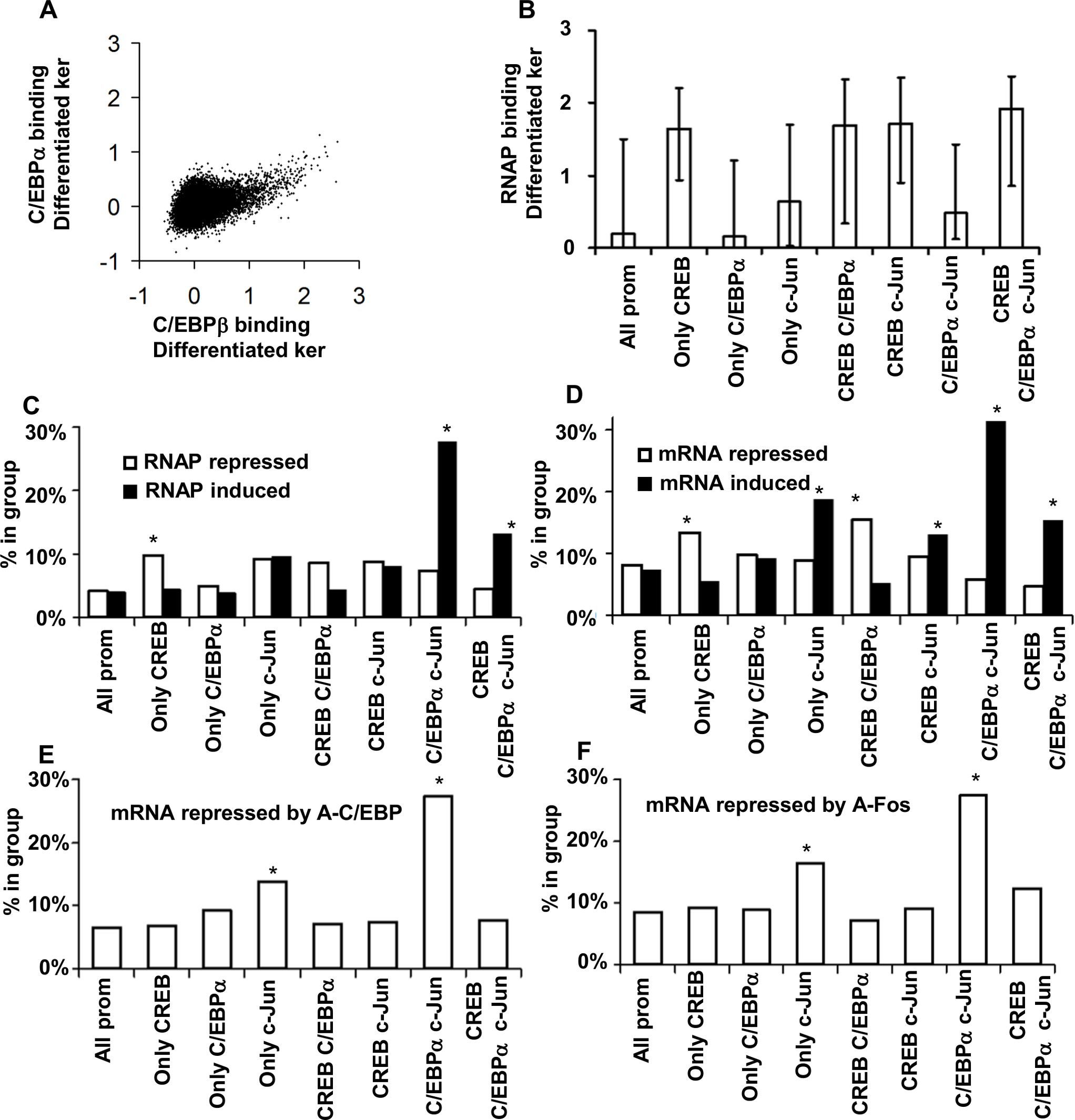

Supplement: Figure S8 — Combinatorial recruitment of CREB, C/EBPα and c-Jun determines activation of promoters in keratinocyte differentiation. A. Scatterplot of C/EBPα binding v.s. C/EBPβ binding show that C/EBOa was detected on a subset of promoters bound by C/EBPβ. B. RNAP binding percentiles (15%, 50% and 85%) in promoters bound by different combinations of CREB, C/EBPα and c-Jun. C. Fraction of promoters bound by different combination of transcription factors in differentiated keratinocytes where RNAP binding is repressed (white bars) or induced by differentiation (black bars). D. Fraction of genes with mRNA levels induced or repressed by differentiation more than 1.4 times in groups of promoters bound by different combinations of transcription factors. * - values are different from expected (p<0.05). ). E. Fraction of genes repressed by A-C/EBP differentiated keratinocytes in groups of promoters bound by different combinations of transcription factors. F. Fraction of genes repressed by A-Fos in differentiated keratinocytes in groups of promoters bound by different combinations of transcription factors * - numbers are different from expected (p<0.001). (TIF) [file pone.0078179.s008.tif]
